# Refractory Hyperinsulinemic Hypoglycemia as a Complication of Roux-en-Y Gastric Bypass Surgery

**DOI:** 10.7759/cureus.69037

**Published:** 2024-09-09

**Authors:** Tawana Mazhude, Tasneem Zahra

**Affiliations:** 1 Internal Medicine, Lincoln Medical Centre, New York, USA; 2 Endocrinology and Diabetes, Lincoln Medical Centre, New York, USA

**Keywords:** adult nesidioblastosis, bariatric surgery complications, hyperinsulinemic hypoglycemia, hypoglycemia, nesidioblastosis, refractory hypoglycemia, reversal bariatric surgery, roux-en-y gastric bypass surgery

## Abstract

This case involves a 45-year-old woman with severe obesity who underwent Roux-en-Y gastric bypass (RYGB) surgery. After one year, she developed daily episodes of severe hypoglycemia, presenting with symptoms of palpitations, diaphoresis, and syncope. The patient was diagnosed with endogenous hyperinsulinemic hypoglycemia, a condition characterized by abnormally high insulin levels leading to low blood glucose, commonly associated with insulinoma. In rare instances, this can be due to nesidioblastosis, an overgrowth of pancreatic beta cells, which is more prevalent in individuals who have undergone bariatric surgery. Diagnostic evaluations included blood tests, abdominal computed tomography and magnetic resonance imaging, continuous glucose monitoring, and hepatic venous sampling to exclude insulinoma. This report details the diagnosis and unsuccessful treatment of endogenous hyperinsulinemic hypoglycemia following RYGB surgery. Interventions included dietary modifications (small, frequent, low-carbohydrate meals), medical management with acarbose 100 mg three times daily, diazoxide 150 mg three times daily, verapamil 40 mg twice daily, and surgical reversal of the RYGB. Ultimately, a percutaneous gastrostomy tube was placed for 24-hour continuous parenteral feeding. Despite these extensive treatment efforts, the patient continues to experience frequent hypoglycemic episodes four years after the bariatric procedure.

## Introduction

Hypoglycemia is a common complication of Roux-en-Y gastric bypass (RYGB) surgery, with most cases being due to late dumping syndrome and resolved through dietary modifications. However, in some cases, hypoglycemia becomes refractory due to endogenous hyperinsulinism [1].

Nesidioblastosis is a form of hyperinsulinemic hypoglycemia and was first described as a complication of RYGB surgery in 2005 [1]. Patients typically present at least one year following the bariatric procedure, with symptoms of hypoglycemia occurring one to three hours after eating. The mechanism of nesidioblastosis after gastric bypass surgery is poorly understood but is thought to involve islet cell hyperplasia and hypertrophy, which can be attributed to elevated serum levels of incretins such as glucagon-like peptide-1 [2,3].

As part of the workup, it is important to exclude common pathologies, such as adrenal insufficiency, exogenous insulin use, and insulinoma, before committing to the diagnosis of hyperinsulinemic hypoglycemia. In an episode of physiologic hypoglycemia, insulin levels are expected to be suppressed. As such, hyperinsulinemic hypoglycemia can be confirmed in patients with symptomatic hypoglycemia (glucose ≤55 mg/dL), inappropriately elevated plasma insulin (>3 uU/mL), elevated C-peptide levels (>0.6 ng/mL), and a negative sulfonylureas screen [1,4].

Nesidioblastosis typically requires histopathologic confirmation, which would demonstrate pathologic overgrowth of pancreatic beta cells. Imaging and radiologic localization procedures, such as the selective arterial calcium stimulation test (SACST) with hepatic venous sampling, must be performed to exclude insulinoma. 

Approximately 228,000 individuals undergo bariatric surgery every year [5], and nesidioblastosis following an RYGB procedure is diagnosed in approximately 0.1%-0.3% of all patients. Adult nesidioblastosis is thought to have a higher incidence in those who have had bariatric surgery than those who have not [3,6].

## Case presentation

A 45-year-old woman with a medical history of well-controlled asthma and obesity with a body mass index of 49 kg/m^2^ underwent a bariatric RYGB procedure. One year after the operation, the patient had lost 150 lb and was hospitalized due to severe postprandial hypoglycemia associated with blood glucose as low as 39 mg/dL at one to two hours after eating. She reported daily episodes of diaphoresis, blurry vision, and dizziness; these were relieved by drinking juice for one month preceding her first admission to the hospital for hypoglycemia.

The patient underwent assessment for all causes of hypoglycemia, including measurements of glucose, insulin, C-peptide (Table 1), serum sulfonylurea screening during episodes of hypoglycemia, and toxicology (Table 2); all results were negative. Hypoglycemia secondary to malnutrition, critical illness, medication, and alcohol intoxication were also excluded at this time. A preliminary diagnosis of dumping syndrome was made, and the patient was discharged on acarbose 50 mg three times daily, advised to avoid meals high in carbohydrates, and given a continuous glucose monitoring device to track blood sugar.

**Table 1:** Diagnostic testing for hyperinsulinemic hypoglycemia

Lab test	Time of result	Result
Blood glucose (74–109 mg/dL)	12/19/21; 10:03	50 mg/dL
C-peptide (1.1–4.4 ng/mL)	12/19/21; 10:03	3.0 ng/mL
Insulin random (2.6–24.9 uU/mL)	12/19/21; 10:03	6.4 uU/mL

**Table 2:** Tests to exclude alternative causes of hypoglycemia

Lab test	Reference values	Result
Insulin autoantibody	<5.0 uU/mL	<5.0
Serum sulfonylurea screen	N/A	Negative
Cosyntropin stimulation test	1 h cortisol level 3–21 ug/dL	26.1
Urine toxicology	N/A	Negative

After 3 months of treatment, the hypoglycemia still did not resolve; therefore, the acarbose dose was doubled to 100 mg and diazoxide 50 mg three times daily was initiated. During the patient's many hospitalizations for hypoglycemia, she underwent abdominal computed tomography, magnetic resonance imaging, and hepatic venous insulin concentration sampling after a selective splanchnic arterial calcium infusion (Table 3), none of which were significant for insulinoma. A cosyntropin test was performed and had an adequate cortisol response, ruling out adrenal insufficiency.

**Table 3:** Selective arterial calcium stimulation test with hepatic venous sampling to exclude insulinoma The selective arterial calcium stimulation test is performed to identify and localize an insulinoma. Samples are drawn from the right hepatic vein after 2 mEq of 10% calcium gluconate is injected into several arteries. Insulin samples are collected prior to calcium stimulation as a baseline, as well as at 30-second, 60-second, 90-second, and 120-second intervals after stimulation of insulin secretion. A diagnosis of insulinoma would see a fourfold increase of insulin from the baseline [7], which localizes to a specific artery. However; this was not the case in the patient.

Location of Sampling	Insulin Time (s)	Insulin Level (uU/mL)
Superior mesenteric artery	0	13.1
	60	15.2
	90	14.6
	120	11.3
Gastroduodenal artery	0	5.4
	30	5.9
	60	12.3
	90	11.2
	120	9.1
Proximal splenic artery	0	9.6
	30	9.6
	90	10.5
	120	6.9
Distal splenic arteries	0	5.6
	30	7.3
	60	5.3
	90	4.3
	120	4.8

The patient subsequently returned to the bariatric surgery clinic and was briefly started on verapamil 40 mg twice daily. However, the patient could not tolerate the medication due to nausea and dizziness. At that time, a decision was made to place a gastrostomy (G) tube for tube feedings to supplement six daily meals, acarbose, and diazoxide. Although the frequency of nocturnal hypoglycemia decreased, the patient continued to have daily episodes of symptomatic hypoglycemia ranging from 40 to 60 mg/dL, and a decision was finally made to remove the G tube and reverse the RYGB. This occurred two years after the initial bariatric procedure.

Despite maximal medical therapy and the reversal of the RYGB, the patient required six inpatient admissions for hypoglycemia within a three-month period. At this time, dumping syndrome was ruled out, and the G tube was replaced to resume tube feeding. The selection of formula as well as the rate of parenteral feeding were carefully coordinated with nutrition services to avoid excessive abdominal pain and nausea associated with excess feeding while remaining sufficient to decrease episodes of hypoglycemia. The dietary recommendations included Jevity 1.2 kcal/ml (Abbott Laboratories, Green Oaks, Illinois, US), which is a high fiber, high protein formulation to be infused at 10 ml/hour, with 6 “mixed meals” a day, where complex carbohydrates are consumed with protein and healthy fats. The course was further complicated by the dislodgement and infection of the G tube on multiple occasions, resulting in additional inpatient admissions.

Four years post-RYGB, the patient is currently being managed with acarbose 100 mg three times daily, diazoxide 150 mg three times daily, and glucagon 1 mg as needed, with 24-hour continuous parenteral feeding at 70 ml/hour via a G tube and frequent low carbohydrate mixed meals. The patient has continued using glucagon injections 4-6 times per month for blood glucose levels ranging from 20 to 50 mg/dL, with a continuous blood glucose monitoring device still demonstrating hypoglycemia 10% of the time. The patient always carries glucose gel and glucagon and must carry a bag containing her feeding supplies whenever she leaves the house. Before the bariatric surgery, she worked as a home health aide, but now she is unable to work due to chronic fatigue, weakness, and syncopal episodes.

## Discussion

Hypoglycemia following bariatric surgery is a common complication. Typically, due to a decreased gastrointestinal (GI) transit time, the patients have an earlier peak of serum glucose, which causes a rapid increase in incretins and insulin levels, resulting in postprandial hypoglycemia [8]; this is known as late dumping syndrome. Treatment is initially aimed at diet modification, introducing small frequent meals that have complex carbohydrates and are high in protein and healthy fats, which helps reduce the exaggerated insulin response.

Should diet modification fail, medical management is initiated with acarbose, which slows the GI transit time and breakdown of carbohydrates. Another medication that is used in the case of elevated insulin is diazoxide, which inhibits insulin secretion from beta cells via the hyperpolarization of the cell membrane. Calcium channel blockers, such as verapamil and nifedipine, have also successfully treated endogenous hyperinsulinemic hypoglycemia [9] by inhibiting calcium-stimulated insulin secretion.

Before reversing the RYGB, a G tube is sometimes placed as a trial to determine whether a reversal will be successful in reducing the severity of symptoms. Feeding through the gastric remnant pouch restores the anatomy prior to the RYGB by slowing the GI transit time and reducing the postprandial serum glucose surge and insulin response [8]. If symptoms decrease, this is considered a good indication that symptoms will improve with reversal. Patients will see a resolution of hypoglycemia following reversal of the RYGB in 88% of cases [10]. This is a rare case where the patient has had no response to any of the aforementioned therapies.

The etiology of hyperinsulinemic hypoglycemia in this patient is unclear and likely multifactorial. There are many hypotheses for the causes of post-bariatric surgery hypoglycemia, including delayed cessation of insulin secretion and decreased ability to suppress insulin secretion and diffuse beta cell hypertrophy, as seen in nesidioblastosis [1,3,8]. Another proposal is that elevated insulin levels due to islet cell hyperfunction lead to the development of severe obesity and its effect on serum glucose is exposed after bariatric surgery.

Selective arterial calcium stimulation testing is a technique used to diagnose insulinoma and has also been found useful in the diagnosis of nesidioblastosis. An insulinoma would cause a fourfold increase in insulin from baseline in the artery supplying the lesion whereas nesidioblastosis would have an increase in insulin across all arteries. Although the SACST result is negative in this patient, this would not be the first case where nesidioblastosis is diagnosed with a positive biopsy and negative SACST [11]. Despite there being no histopathologic confirmation of nesidioblastosis, it remains high on the differential diagnosis, as all other causes of hypoglycemia have been excluded.

One last available therapy is partial pancreatectomy, where an effective decrease in beta cell mass reduces the amount of insulin secreted by the pancreas. However, the degree of resection required is not clear, which makes the procedure high risk with an uncertain likelihood of success; up to 46% of patients have a recurrence of hypoglycemia after pancreatectomy [10,12].

## Conclusions

As the prevalence of obesity and patients undergoing bariatric surgery increases, hypoglycemia as a complication is now being more frequently documented. Adult nesidioblastosis is uncommon and appears to have a higher incidence in patients who have had RYGB surgery. Although a causal relationship has not been established, it must be considered a possible complication of the procedure when a patient presents with postprandial hypoglycemia and inappropriately elevated insulin levels in the absence of insulin antibodies and insulinoma. Further research is needed to identify the pathophysiology of hyperinsulinemic hypoglycemia so that more targeted treatments can be developed. Currently, a diagnosis of nesidioblastosis cannot be achieved without invasive procedures, which contributes to its low rate of diagnosis.
